# Stacking of doxorubicin on folic acid-targeted multiwalled carbon nanotubes for *in vivo* chemotherapy of tumors

**DOI:** 10.1080/10717544.2018.1501120

**Published:** 2018-10-22

**Authors:** Yan Yan, Ruizhi Wang, Yong Hu, Rongyue Sun, Tian Song, Xiangyang Shi, Shimeng Yin

**Affiliations:** a Department of Obstetrics, Shanghai First Maternity and Infant Hospital, Tongji University School of Medicine, Shanghai, P. R. China;; b Department of Radiology, Huadong Hospital, Fudan University, Shanghai, P. R. China;; c College of Chemistry, Chemical Engineering and Biotechnology, Donghua University, Shanghai, P. R. China

**Keywords:** Multiwalled carbon nanotubes, polyethyleneimine, doxorubicin, folic acid, tumor targeting

## Abstract

In this work, we developed a novel active targeting and pH-responsive system for delivering the drug doxorubicin (DOX) to tumor sites using folic acid (FA)-modified multiwalled carbon nanotubes (MWCNTs). Acid-treated MWCNTs with carboxyl groups were first covalently conjugated with polyethyleneimine (PEI). Subsequent sequential modification with FA (via a polyethylene glycol spacer), fluorescein isothiocyanate (FI), and acetic anhydride/triethylamine resulted in multifunctional FA-bound MWCNT (MWCNT-PEI.Ac-FI-PEG-FA) nanomaterials that possessed exceptional colloidal stability and good biocompatibility in a given concentration range. The FA-bound MWCNTs were characterized using various techniques and exhibited a high drug loading and an encapsulation efficiency as high as 70.4%. DOX/MWCNT-PEI.Ac-FI-PEG-FA nanocomplexes (DOX/MWCNT NCs) exhibited pH-responsive release in acidic environments. Importantly, the DOX/MWCNT NCs targeted tumor cells overexpressing FA receptors (FARs) and effectively inhibited their growth. *In vivo* anticancer experiments demonstrated that DOX/MWCNT NCs not only enhanced the suppression of tumor growth but also decreased the side effects of free DOX. The developed FA-modified MWCNTs with an unconventionally high DOX loading boosted *in vivo* anti-tumor efficacy, and the lower systemic toxicity may be utilized for tumor therapy upon clinical translation.

## 1. Introduction

Doxorubicin (DOX), a typical anthracycline-class chemotherapeutic agent, is widely used in clinics for the chemotherapy of various cancers, including hematological malignancies, lymphoma, and many other types of solid tumors. Because of the serious disadvantages associated with free DOX, including its salient cytotoxicity to normal cells, water insolubility, and ease in being removed by the blood stream (Wang et al., 2011), a better system to load and release DOX is urgently needed (Tahover et al., 2015). In recent years, various nanoscale delivery systems have been developed as novel carriers, for example microcapsules (Du et al., 2013; Liu et al., 2014), liposomes (Tahover et al., 2015), polymer micelles (Movassaghian et al., 2015), mesoporous silica nanoparticles (MSNs) (Hai et al., 2015; Qu et al., 2015), dendrimers (Kesharwani et al., 2014), chitosan (Assa et al., 2017), and other nanomaterials.

Carbon nanotubes (CNTs) have emerged as an attractive nanocarrier due to their well-known merits, such as their high surface areas, ultralow weights, excellent chemical and thermal stability, and outstanding capability to cross cell membranes (Shi Kam et al., 2004; Feazell et al., 2007; Hersam, 2008). However, their water insolubility hinders the biomedical applications of CNTs. A common route via which to render CNTs water soluble is the functionalization of CNTs with polymers or small molecules (Prato et al., 2008; Bhirde et al., 2009), which improves the biocompatibility of CNTs (Chłopek et al., 2006; Lacerda et al., 2006). Liu et al. (2007) prepared phospholipids bearing polyethylene glycol (PEG)-functionalized single-walled CNTs (SWCNTs) by noncovalent modification. PEGylated SWCNTs are quite stable *in vivo* and have a longer blood circulation time and lower uptake by the reticuloendothelial system (RES). Liu et al. (2009) further fabricated DOX-stacked PEGylated SWCNTs by attaching DOX to the surface of the PEGylated SWCNTs through π–π stacking interactions. However, exploring the covalent modification of CNT nanostructures as stable nanoplatforms for drug loading and the chemotherapy of tumors is still challenging.

We have previously shown that dendrimer- and polyethyleneimine (PEI)-modified multiwalled CNTs (MWCNTs) are easily prepared, allowing various biofunctionalizations to occur (Wen et al., 2013; Cao et al., 2015). We accordingly hypothesize that dendrimer- or PEI-modified MWCNTs may also adsorb DOX through π–π stacking interactions, thereby providing DOX-loaded MWCNT nanocomplexes (NCs) as chemotherapeutic agents for cancer. Unfortunately, most of these contrast or therapeutic agents suffer from poor delivery without targeting ligands, leading to the accumulation of only small amounts of injected drugs in target tissues (Zerda et al., 2012). Our previous studies demonstrated that PEI-based nanomaterials can be easily modified with the targeting ligands folic acid (FA), arginine–glycine–aspartate (RGD), and hyaluronic acid (HA) for the specific recognition of tumors that highly express FA receptor (FAR) (Hu et al., 2016), α_v_β_3_ integrin (Cao et al., 2015), and CD44 receptor (Hu et al., 2015), respectively. The multifunctional MWCNTs designed in this study were expected to be coupled with PEI for further modification with targeting ligands, hence creating a novel system to load and release DOX for precise treatment of cancer.

**Scheme 1.**
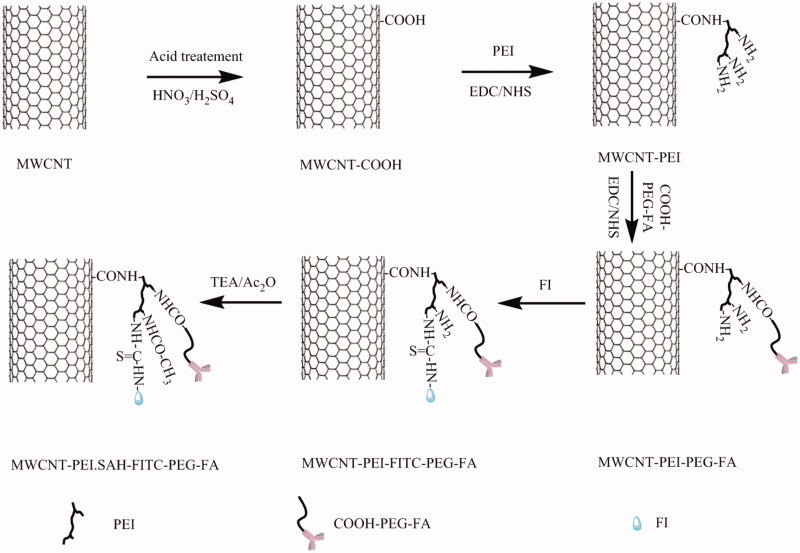
Schematic representation of the synthesis of the DOX/MWCNT-PEI.Ac-FI-PEG-FA nanocomplexes.

In this work, we designed a novel drug delivery system based on FA-modified DOX-loaded MWCNT NCs for the chemotherapy of tumors. First, acid-treated MWCNTs were grafted with PEI in a manner similar to that previously reported (Shen et al., 2009). After the conjugation of PEI, the resultant MWCNT-PEI nanomaterials were utilized to prepare multifunctional MWCNT-PEI.Ac-FI-PEG-FA (FA-targeted MWCNT) nanomaterials by successive modification with COOH-PEG-FA and fluorescein isothiocyanate (FI) and the surface neutralization of excess PEI terminal amines by using acetic anhydride. Ultimately, as-prepared FA-targeted MWCNT nanomaterials were used as nanocarriers to carry DOX via π–π stacking interactions (Scheme 1). The multifunctional DOX/MWCNT-PEI.Ac-FI-PEG-FA (DOX/MWCNT) NCs and their intermediate products were characterized in detail. The *in vitro* hemocompatibility/cytocompatibility and the specific recognition of FAR-overexpressing cancer cells by FA-targeted MWCNT nanomaterials were evaluated. After the nanomaterials were loaded with DOX, their potential for *in vivo* targeted chemotherapy of tumors was evaluated in detail.

## 2. Experimental

### 2.1. Materials

The MWCNTs (diameter = 30–70 nm, length = 100 nm–2 μm) were synthesized and characterized as described in previous reports (Petersen et al., 2008a; Petersen et al., 2008b). Amine-PEG-carboxymethyl (NH_2_-PEG-COOH, MW = 2000) was supplied by Shanghai Yonyi Biotechnology Corporation (Shanghai, China). N-hydroxysuccinimide (NHS), ethyl(dimethylaminopropyl) carbodiimide (EDC), FA, and FI were purchased from J&K Chemical Ltd (Shanghai, China). DOX (the term ‘DOX’ refers to DOX·HCl unless otherwise stated) was supplied by Beijing Huafeng Pharmaceutical Co., Ltd (Beijing, China). PEI (MW = 25,000), acetic anhydride, triethylamine, dimethyl sulfoxide (DMSO), and all the other chemicals and solvents were supplied by Sigma-Aldrich (St. Louis, MO). All of the chemicals were used without further purification. HeLa cells (a human cervical cell line) were obtained from the Institute of Biochemistry and Cell Biology (Chinese Academy of Sciences, Shanghai, China). Fetal bovine serum (FBS), Dulbecco’s modified eagle medium (DMEM), penicillin, and streptomycin were all supplied by Hangzhou Jinuo Biomedical Technology (Hangzhou, China). Cell counting kit-8 (CCK-8) was supplied by 7Sea Pharmatech Co., Ltd (Shanghai, China). Water with a resistivity exceeding 18.2 MΩ.cm was used in all experiments and had been passed through a Milli-Q Plus 185 water purification system (Millipore, Bedford, MA). Regenerated cellulose dialysis membranes with molecular weight cutoffs (MWCOs) of 1000, 2000, and 50,000 Da were supplied by Fisher (Pittsburgh, PA).

### 2.2. Fabrication of multifunctional FA-modified MWCNT nanomaterials

The multifunctional FA-modified MWCNTs were prepared as described in our previous work (Hu et al., 2016). To adorn the surface of MWCNTs with carboxylic acid residues, the pristine MWCNTs were treated with concentrated HNO_3_/H_2_SO_4_ (v/v = 3:1) for 2 h, then filtered and dried (Shi et al., 2009). The route to chemically bond PEI to the acid-treated MWCNTs was borrowed from the previous protocol with small changes (Shen et al., 2009). Briefly, the acid-treated MWCNTs (80 mg) were activated by EDC (80 mg) and NHS (48 mg) in DMSO (80 mL) for 3 h with vigorous magnetic stirring to fully activate the carboxyl groups on the MWCNTs. PEI, with an abundance of primary amines and a hyperbranched structure, was then used as a modifier to improve the water stability and modifiability of MWCNTs. A DMSO PEI solution (80 mg, 20 mL) was typically added dropwise into the above mixture under magnetic stirring. After 3 days, the reaction mixture was dialyzed against phosphate-buffered saline (PBS) (3 times, 2 L) and deionized water (6 times, 2 L) for 3 days with a dialysis membrane (MWCO = 50,000) and lyophilized to acquire MWCNT-PEI.

Attaching FA via a PEG spacer allows the nanoplatform to bind FAR-overexpressing cancer cells with a high affinity for biomedical applications (Li et al., 2013). Covalent immobilization of COOH-PEG-FA onto MWCNT-PEI via the formation of an amido bond is therefore desirable. In brief, COOH-PEG-FA (40 mg), fabricated according to our previous report (Hu et al., 2016), was activated by EDC (31 mg) and NHS (19 mg) in DMSO (20 mL), and this reaction was complete after 3 h. Activated COOH-PEG-FA was then added dropwise to the above-obtained MWCNT-PEI (54.5 mg) DMSO solution (20 mL). After 3 days of thorough magnetic stirring, the raw product was purified by dialysis with a dialysis membrane (MWCO = 50,000) and lyophilized to obtain MWCNT-PEI-PEG-FA.

The FI was selected as an amine-reactive fluorescent probe with which to label MWCNTs. Briefly, a DMSO solution (1 mL) containing FI (3.2 mg) was reacted with the MWCNT-PEI-PEG-FA (58.9 mg redispersed in 20 mL of DMSO), and then the obtained mixture was stirred for 24 h while shielded from light at room temperature. MWCNT-PEI-FI-PEG-FA was purified by dialysis with a dialysis membrane (MWCO = 50,000) and lyophilized to obtain a powder, which was then redispersed in water and reacted with triethylamine (45 μL) for 30 min. Acetic anhydride (31 μL) was then added dropwise to the reaction mixture to neutralize the remaining PEI terminal amines, and the mixture was stirred vigorously for another 24 h. A dialysis membrane (MWCO = 1000) was used to remove the excess reactants and byproducts. Negatively charged FA-targeted MWCNTs were obtained after lyophilization. The multifunctional FA-targeted MWCNTs were stored in water or PBS for further experiments.

### 2.3. Characterization techniques

Transmission electron microscopy (TEM) images were taken on an electron microscope (JEOL 2010 F, Tokyo, Japan) at an operating voltage of 200 kV. The sample, an FA-modified MWCNT suspension in water (5 μL, 0.1 mg/mL), was deposited onto a carbon-coated copper mesh grid and allowed to dry naturally at room temperature. The zeta potential was measured with a Malvern Zetasizer-Nano Series-Zen 3600 (Worcestershire, UK). At least 10 measurements were acquired for each sample. Thermal gravimetric analysis (TGA) was performed using a TG 209 F1 (NETZSCH Instruments Co., Ltd, Selb/Bavaria, Germany) thermal gravimetric analyzer. The sample was heated from ambient temperature to 900 °C at a rate of 10 °C/min in a nitrogen environment. A Lambda 25 UV-Vis spectrophotometer (PerkinElmer, Boston, MA) was used to measure the UV-Vis spectra of the samples.

### 2.4. Preparation of DOX-loaded MWCNT NCs

According to previous reports (Wen et al., 2013), the surface of CNTs can be attached to DOX through π–π stacking interactions. FA-targeted MWCNTs (10 mg) dispersed in 5 mL of water were mixed with an aqueous solution of DOX (1 mg/mL, 5 mL). The pH value of the solution was adjusted to 9.5, and the mixture was kept at room temperature in dark for 24 h with stirring. Unbound DOX molecules were removed from the mixture by centrifugation (13,000 rpm, 10 min) and redispersed in water several times until the supernatant had no absorbance at wavelengths shorter than 481 nm. The purified multifunctional DOX/MWCNT-PEI.Ac-FI-PEG-FA (DOX/MWCNT) NCs were lyophilized and kept at −20 °C while shielded from light before use. The mixture was centrifuged, and the supernatants were all collected to quantify the unbound DOX by comparing the supernatant absorbance at 481 nm, as determined by UV-Vis spectrometry. A calibration curve of absorbance vs. DOX concentration that had been obtained under the same conditions was used. DOX loading efficiency and loading percentage were calculated using Equations (1) and (2), respectively:

(1) $$\text{Loading}\;\text{efficiency}\;=\;(M_{1}/M_{0})\times \;100\%$$

(2) $$\text{Loading}\;\text{percentage}\;=\;(M_{1}/\left(M_{1}+M_{2}\right))\times 100\%$$

where M_0_, M_1_, and M_2_ represent the masses of the initial DOX, the loaded DOX, and the FA-targeted MWCNTs, respectively.

### 2.5. *In vitro* study of drug release kinetics

To determine the amount of DOX released from the DOX/MWCNT NCs, we dispersed the complexes (2 mg) in 1 mL of PBS (pH 7.4) or acetate buffer (pH = 5.0), kept this mixture in a dialysis bag with a MWCO of 2000 Da, and performed dialysis in the corresponding buffer (9 mL). This process was performed with three replicates. All samples were cultivated in a 37 °C humidified constant temperature shaker. At each particular point in time, 1 mL of buffer was removed from the outside of the corresponding buffer, and UV-Vis spectroscopy was used to quantify the concentration of the released DOX. The volume of the outside buffer medium was kept constant by resupplying 1 mL of the corresponding buffer.

### 2.6. Cytotoxicity assays and cell morphology observation

HeLa cells were routinely cultivated and passaged in 25 cm^2^ plates with regular DMEM (10% heat-inactivated FBS and 1% penicillin/streptomycin) at 37 °C and a 5% CO_2_ humid environment (Sun et al., 2006). Cells not receiving a pretreatment of free FA and thus overexpressing FAR were termed HeLa-HFAR. Cells that received a pretreatment of free FA (2.5 μM) were cultivated for 24 h so they had decreased expression of FAR and were then termed HeLa-LFAR. Unless otherwise specified, the term ‘HeLa cells’ refers to HeLa-HFAR cells.

CCK-8 cell viability assays were employed to determine the treatment effect of the DOX/MWCNT NCs using standard manufacturer’s instructions. Briefly, HeLa cells at a density of 1 × 10^4^ cells per well were seeded in a 96-well plate. After being cultivated with 200 μL of DMEM at 37 °C and 5% CO_2_ for 12 h, the adherent cells were incubated with 200 μL of DMEM containing free DOX and DOX/MWCNTs with identical DOX concentrations (0, 0.5, 1, 2, and 4 mg/L). FA-targeted MWCNTs without DOX were used at the same concentration as the corresponding FA-targeted DOX/MWCNT NCs. After the sample was incubated for another 24 h, 20 μL of CCK-8 was added to each well, and the HeLa cells were then cultivated for 4 h in the same environment. The absorbance of all samples was then determined at 450 nm using a Thermo Scientific Multiskan MK3 ELISA reader (Thermo Scientific, Hudson, NH). Cell viability was calculated according to Equation (3):

(3) $$\text{Cell}\;\text{viability}\;=\;\frac{A_{1}-B}{A_{0}-B}\times 100\%$$

where A_0_ and A_1_ represent the OD values obtained when CCK-8 was used for DMEM containing HeLa cells after the cells had been treated with PBS and multifunctional FA-targeted MWCNTs, respectively. B represents the OD value obtained when CCK-8 was used for DMEM alone. The average and standard deviation (SD) of five parallel measurements of all samples were recorded.

After HeLa cells were pretreated with FA-targeted MWCNTs and DOX/MWCNTs, an inverted phase contrast microscope (Leica DM IL LED) was applied to observe their morphology. The magnification was set at 200× for all samples.

### 2.7. *In vitro* specific cellular uptake assay

Flow cytometry was performed to quantitatively evaluate the specific uptake of the DOX/MWCNT NCs by HeLa-HFAR cells. Both HeLa-HFAR and HeLa-LFAR cells were seeded in 12-well culture plates at a density of 2 × 10^5^ cells/well. After the adherent cells were incubated overnight in 1 mL of DMEM at 37 °C and 5% CO_2_, the medium was carefully aspirated and replenished with 1 mL of fresh medium containing DOX/MWCNT NCs with the same concentration of DOX (2 mg/L) and PBS (control). After another 2-h incubation, the cells were successively rinsed with PBS 5 times, harvested (by trypsinization and centrifugation), and resuspended in 1 mL of PBS. A FACSCalibur flow cytometer (Becton Dickinson, Franklin Lakes, NJ) was then employed to analyze the fluorescence signal intensity. The FI fluorescence of 10^4^ cells was estimated, and the measurement was repeated 3 times.

The specific uptake of DOX/MWCNT NCs by HeLa-HFAR cells was further examined by confocal microscopy (Carl Zeiss LSM 700, Jena, Germany). Briefly, cover slips were first treated and fixed in a 12-well plate according to a previously described method (Wang et al., 2013). HeLa-HFAR cells were then seeded in the culture plate at a density of 2 × 10^5^ cells per well with 1 mL of fresh medium and cultured overnight at 37 °C and 5% CO_2_ to leave the cell wells attached to the cover slips. The medium was then replaced with 1 mL of fresh medium containing PBS (control) and the DOX/MWCNT NCs with 2 mg/L DOX. After a 4-h incubation at 37 °C and 5% CO_2_, the cells were rinsed with PBS 3 times, fixed with glutaraldehyde (2.5%) for 15 min at 4 °C, and counterstained with 4',6-diamidino-2-phenylindole (DAPI, 1 μg/mL) for 15 min at 37 °C. Images of the cells attached to the cover slips were collected by using a 63× oil immersion objective lens. HeLa-LFAR cells were also handled as described above.

### 2.8. *In vitro* targeted cancer cell inhibition

To evaluate the inhibitory effect of the DOX/MWCNT NCs on the targeted cancer cells, both HeLa-HFAR and HeLa-LFAR cells were seeded in a 96-well plate at a density of 1 × 10^4^ cells/well in 200 μL of medium and cultivated overnight at 37 °C and 5% CO_2_. The solution was then replaced with fresh medium containing DOX/MWCNT NCs with a DOX concentration of 2 mg/L. After 2 h of cultivation, the cells were rinsed 3 times with PBS and cultivated in fresh DMEM for another 48 h. Finally, CCK-8 assays were used to quantify the cells according to the protocols described above.

### 2.9. *In vivo* targeted cancer cell inhibition

Animal experiments were approved by the corresponding ethical committee, and animal experimental ethics guidelines were followed. Six-week-old female BALB/c nude mice (22–26 g, Shanghai Slac Laboratory Animal Center, Shanghai, China) were subcutaneously injected with 2 × 10^6^ HeLa cells/mouse in the right flank. When the volume of tumor nodules reached approximately 1.0 cm^3^, mice were randomly allocated into four equal groups (*n* = 3) and subjected to different treatments. Namely, the mice were regularly fed without any treatment (control group), intravenously delivered DOX (5 mg/kg) (DOX group), intravenously delivered FA-targeted MWCNT (14.2 mg/kg) (MWCNT group), or intravenously delivered DOX/MWCNT NCs ([DOX] = 5 mg/kg, in 0.1 mL of PBS) (DOX/MWCNT group). Treatments were repeated on days two, four, and seven after the initial treatment.

The body weights of all mice and the sizes of the tumors were recorded on the days mentioned above. The tumor volume was calculated according to the equation V = $\frac{1}{2}$ (tumor length × (tumor width)^2^). Tumors were photographed *ex vivo* on day 30 post-treatment.

### 2.10. Histological examination

To further investigate the mechanisms underlying *in vivo* anti-tumor and chemotherapeutic effects at the histological level, we subjected four groups of mice bearing HeLa tumors to different treatments as described in the procedures above. The mice were euthanized at 48 h after treatment, and the tumors were surgically removed, formalin-fixed, paraffin-embedded, and serially cut for hematoxylin–eosin (H&E) staining (Zhu et al., 2014). The embedded sections were stained with TdT-mediated dUTP nick-end labeling (TUNEL) fluorescent dye following the instructions of an In-Situ Cell Death Detection Kit (Roche Applied Science, Shanghai, China). DAPI was used for nuclear counterstaining. The morphology of random stained tumor tissue slices was observed using a Leica DM IL LED inverted phase contrast microscope or a Zeiss inverted fluorescence microscope. The TUNEL-positive cells (apoptotic cells) in each specimen were counted, and the percentages of apoptotic cells were calculated from five random fields of the images.

For *in vivo* toxicity evaluation, the main organs, including the lungs, kidneys, liver, spleen, and heart, of healthy mice were collected on day 14 post-injection of DOX/MWCNT NCs *via* the tail vein. Healthy mice that received no treatment were used as a control. H&E staining was performed again for histological examinations of the main organs.

### 2.11. Biodistribution study

To assess the DOX biodistribution in tumor-bearing mice after IV treatment with DOX/MWCNT NCs, a previously reported protocol was employed (Liu et al., 2009). Briefly, tumor-bearing mice were sacrificed at 12 h after the injection of DOX/MWCNT NCs. The main organs (lungs, kidneys, liver, spleen, heart, and tumor, 0.1–0.2 g of each) were wet-weighed and homogenized in 0.5 mL of lysis buffer (0.25 M sucrose, 40 mM Tris acetate, and 10 mM EDTA) with a low-temperature water bath ultrahigh pressure continuous flow cell disrupter (JN-02c, Guangzhou Juneng Biology & Technology Co., Ltd, Guangzhou, China). To quantify DOX, tissue lysate (200 μL) was mixed with Triton X-100 (10%, 100 μL). After vigorous vortexing, the extraction solution (1 mL, 0.75 M HCl in isopropanol) was added, and the samples were cultivated overnight at −20 °C. After 15 min of centrifugation at 24,000 g, the fluorescence of the supernatant was measured with a BioTek Synergy 2 Multi-Mode Reader (λ_ex_ = 485 nm, λ_em_ = 590 nm). To correct for nonspecific fluorescence, we used control mice without any treatment as a control. Three mice were included.

### 2.12. Statistical analysis

The significance of the experimental data was determined with one-way ANOVA. A value of 0.05 was considered the threshold for significance, and the data were marked as (*) for *p* < .05, (**) for *p* < .01, and (***) for *p* < .001.

## 3. Results and discussion

### 3.1. Synthesis and characterization of multifunctional FA-targeted MWCNTs

The strategy to modify hyperbranched PEI onto carboxylated MWCNT (Shen et al., 2009) allowed us to synthesize FA-targeted MWCNTs for DOX loading. Briefly, MWCNTs were first endowed with carboxylic groups in strongly acidic solutions. PEI, with an abundance of terminal amines, was then grafted onto MWCNTs via EDC coupling chemistry, followed by sequential modifications with COOH-PEG-FA and FI and the acetylation of the remaining amines of PEI via covalent conjugation chemistry to form multifunctional FA-targeted MWCNT NCs.

The TGA (Figure S1a) was applied to characterize the surface modification of the MWCNTs. Because of the acid-treated MWCNTs (MWCNT-COOH), no weight loss occurred when the temperature was increased to 500 °C. In sharp contrast, MWCNT-PEI lost 23.3% of its weight at the same temperature, which was attributed to the existence of the PEI linked to the surface of the MWCNTs. For the samples that had received further modification, an additional 21.6% weight loss was observed when MWCNT-PEI-PEG-FA was heated to the same temperature due to the covalent conjugation of COOH-PEG-FA to terminal PEI amines. The successful modification of COOH-PEG-FA was also verified by UV-Vis spectra (Figure S2). Compared to MWCNT-PEI, MWCNT-PEI-PEG-FA displays an additional UV absorbance peak at approximately 270 nm (Huang et al., 2011). Further, FI modification gives MWCNT-PEI another UV absorbance peak at approximately 505 nm (Cao et al., 2015). Zeta potentials were measured to elucidate the single steps of the surface modification (Figure S1b). Because of the exposed carboxyl groups, MWCNT-COOH clearly had a relatively negative charge, and it had a zeta potential of −40.8 mV. MWCNT-PEI, formed after grafting with PEI, showed a quite positive surface potential (+33.5 mV). However, the zeta potential of MWCNT-PEI decreased to +20.7 mV as some of the terminal amines were consumed by subsequent modification with COOH-PEG-FA and FI. To further improve the biocompatibility of the MWCNTs and avoid nonspecific cell membrane binding (Shen et al., 2009), we acetylated MWCNT-PEI-FI-PEG-FA to generate FA-targeted MWCNTs with a lower positive charge (+11.2 mV). The FA-targeted MWCNTs were ultimately characterized with TEM (Figure S3). Compared to the pristine MWCNTs (Shen et al., 2009), the MWCNTs with successive modifications did not exhibit appreciable morphology changes or aggregation, indicating quite uniform biofunctionalization.

### 3.2. *In vitro* stacking and release of DOX

Under basic conditions, DOX is deprotonated and quite hydrophobic, which enhances its effective π–π stacking interactions with MWCNTs (Wen et al., 2013; Cao et al., 2015). According to the optimal conditions (except at pH = 9.5) reported previously (Wen et al., 2013), the multifunctional DOX/MWCNT NCs were loaded with the anticancer drug DOX. UV-Vis spectroscopy was also performed to characterize the DOX/MWCNT NCs (Figure S4). DOX/MWCNT NCs clearly display an enhanced absorption intensity at 481 nm (characteristic absorption peak of DOX), indicating the success of DOX loading within the MWCNTs. By quantification via a standard calibration curve of DOX absorbance and concentration, DOX loading efficiency and loading percentage were calculated to be 70.4 and 26.0%, respectively. DOX/MWCNT NCs possessed good colloid stability in water, PBS, and cell culture medium, and no precipitation was observed within at least 2 months.

The loaded DOX drug should be released to achieve full therapeutic functionality. We then studied the dissolution curve of DOX release from DOX/MWCNT NCs at two different pH values (pH = 5.0, representing the acidic tumor microenvironment, and 7.4, representing the normal physiological environment). As shown in Figure S5, the DOX/MWCNT NCs released DOX in a pH-responsive manner. In the acidic tumor microenvironment (pH = 5.0), 58.3% of DOX was released in the first 10 h, whereas only 10.1% of DOX was released in the normal physiological environment (pH = 7.4) simultaneously. In the different pH settings of tumors and normal tissue, the DOX release percentages in 24 h were 75.5 and 17.2%, respectively. The prolonged release of DOX from the NCs in the normal physiological environment and the rapid release in the acidic tumor environment will benefit the treatment of tumors with an acidic environment (pH = 5–6).

### 3.3. Therapeutic efficacy of DOX/MWCNT NCs

**Figure 1.**
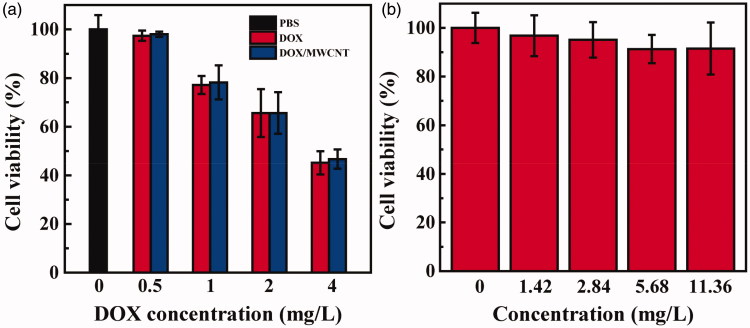
CCK-8 assays of HeLa cells treated for 24 h with free DOX and DOX/MWCNT NCs at DOX concentrations of 0–4 mg/L (a) and CCK-8 assays of DOX-free FA-targeted MWCNTs at concentrations equivalent to those of the DOX/MWCNT NCs with DOX concentrations between 1.42 and 11.36 mg/L (b).

The therapeutic efficacy of DOX/MWCNT NCs was evaluated with HeLa cells. After a 24-h incubation of DOX/MWCNT NCs with cells, a CCK-8 assay was applied to test the cellular viability (Figure 1(a)). Similar to the situation for free DOX, the treatment with DOX/MWCNT NCs caused a dramatic decline in cellular viability compared with that of the control cells with no treatment. HeLa cells cultivated with free DOX and DOX/MWCNT NCs had a viability of 45.2 ± 4.8 and 46.7 ± 4.0% ([DOX] = 4 mg/L), respectively. The IC_50_ values of free DOX and DOX/MWCNT NCs were calculated to be 3.45 and 3.53 mg/L, respectively. These results indicate that the loading of DOX within the NCs does not compromise the anticancer activity of free DOX. To rule out the possible toxicity of the NCs as a reason for these results, FA-targeted MWCNTs without DOX were next evaluated (Figure 1(b)). At the same concentrations, the cell viability was always greater than 90%, indicating that FA-targeted MWCNTs are nontoxic to HeLa cells. A similar morphology was observed for HeLa cells between those treated with FA-targeted MWCNTs at the concentration of 8.4 mg/L (Figure S6b) and those treated with PBS (Figure S6a), indicating that the FA-targeted MWCNTs without DOX negligibly affected the HeLa cells. In contrast, a large portion of HeLa cells treated for 24 h with DOX/MWCNT NCs ([DOX] = 4 mg/L) (Figure S6c) was rounded and detached, implying cell death.

### 3.4. Flow cytometry assays and confocal microscopy

The overexpression of FAR is found in many different types of carcinoma cells, including those of the brain, lung, kidney, colon, breast, ovary, uterus, testis, and myelocytic blood (Low et al., 2008). To provide MWCNTs with targeting ability, we grafted PEGylated FA to the surface of MWCNTs for cancer-targeted delivery of DOX to cells overexpressing high-affinity FAR (Hu et al., 2016).

**Figure 2.**
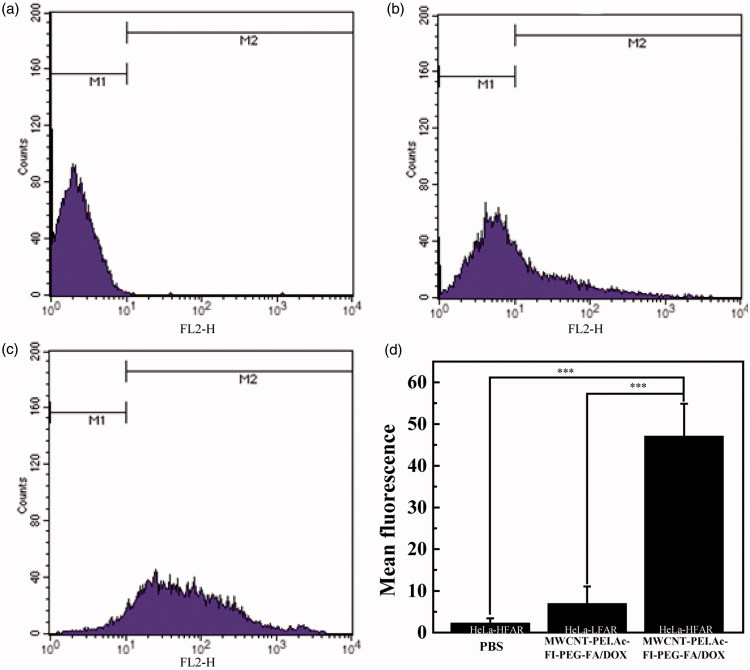
Flow cytometry analysis of HeLa-HFAR (a, c) and HeLa-LFAR (b) after treatment with PBS (a) or DOX/MWCNT NCs at a DOX concentration of 2 mg/L (b,c). The fluorescence (d) is shown as the mean ± SD.

Flow cytometry could be applied to quantify the cellular uptake of DOX/MWCNT NCs via the inherent red fluorescence of DOX after 2 h of cultivation with the NCs. As shown in Figure 2, treatment of HeLa-LFAR cells with DOX/MWCNT NCs (Figure 5(b,d)) slightly increased the fluorescence signal within the cells. In comparison, HeLa-HFAR cells treated with DOX/MWCNT NCs (Figure 5(c,d)) displayed a fluorescence signal significantly greater than that of the PBS control (Figure 5(a,d)). These results indicate that the DOX/MWCNT NCs can be bound to HeLa-HFAR cells only via the FAR-mediated pathway, in agreement with the literature (Li et al., 2015; Hu et al., 2016).

**Figure 3.**
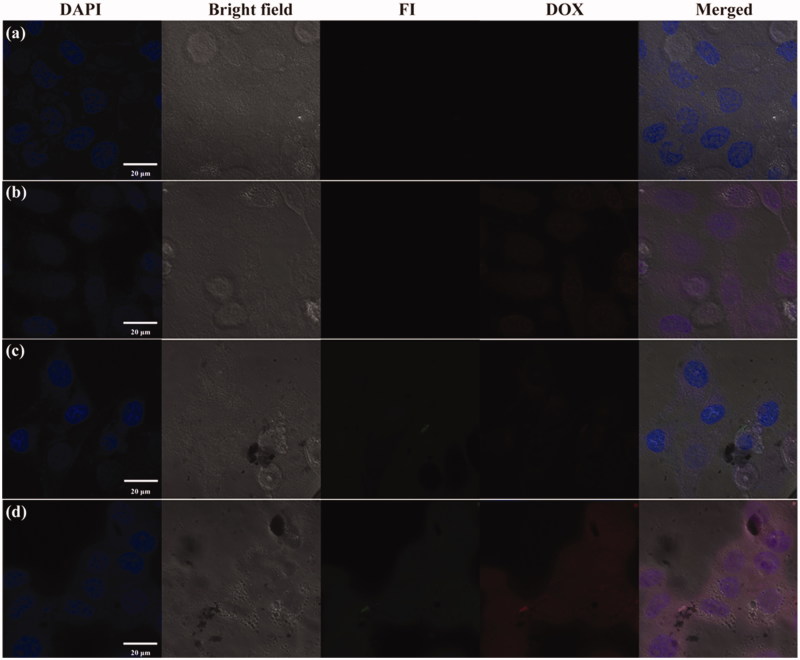
Confocal microscope images of HeLa-HFAR (a,b,d) and HeLa-LFAR (c) cells after treatment with PBS (a), DOX (b), and DOX/MWCNTs (c,d) at a DOX concentration of 2 mg/L for 2 h.

Based on the red fluorescence of DOX and the green fluorescence of FI, the confocal microscope imaging of these probes was used to evaluate the cellular uptake of the DOX/MWCNT NCs. As shown in Figure 3, after a 2-h incubation with the DOX/MWCNT NCs, HeLa-HFAR cells displayed the strongest red and green fluorescence signals, which are closely related to the specific assimilation of the DOX/MWCNT NCs into the cytoplasm of the cells (Figure 3(d)). In contrast, for HeLa-LFAR cells treated with the same NCs (Figure 3(c)) compared with cells treated with PBS (Figure 3(a)) or DOX (Figure 3(b)), no appreciable fluorescence signals were observed. The results suggest that FA can enable MWCNT-mediated targeted delivery of DOX into cancer cells overexpressing FAR.

### 3.5. *In vitro* targeted cancer cell inhibition

To confirm the targeted therapeutic efficacy of the DOX/MWCNT NCs, we replaced the medium with the same volume of DOX-free fresh medium after NCs were cultivated with cells for 2 h, and the cells were then cultivated for 48 h at 37 °C before the CCK-8 assay was performed (Figure S7). After treatment with the DOX/MWCNT NCs, the viability of HeLa-HFAR cells clearly decreased to 54.8%. In sharp contrast, the HeLa-LFAR cells maintained 88.7% cell viability. This result indicates that with FA modification, the DOX/MWCNT NCs could specially inhibit cancer cell survival via FAR-mediated binding, thereby resulting in greater intracellular uptake.

### 3.6. *In vivo* targeted cancer cell inhibition

**Figure 4.**
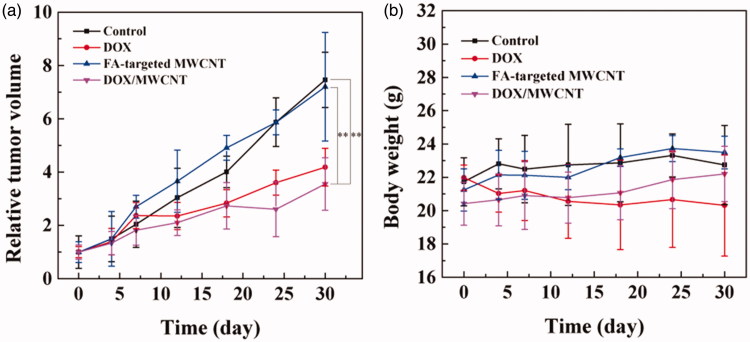
(a) Tumor volume change profiles and (b) body weight changes of tumor-bearing mice (*n* = 3) as a function of time post-treatment.

The promising *in vivo* targeted cancer cell inhibition by the DOX/MWCNT NCs inspired us to further explore their *in vivo* applicability for targeted inhibition of cancer. Figure 4(a) shows that the tumor growth rate of the mice injected with DOX/MWCNT NCs (DOX/MWCNT group) was much slower than that of the mice without any treatment (control group) and of those only treated with either free DOX (DOX group) or FA-targeted MWCNTs (MWCNT group). The volume of tumors treated with normal saline increased by 7.46 ± 1.04 times after 30 days. In contrast, the DOX/MWCNT group showed an obvious tumor suppression effect (3.55 ± 0.99 times increase in tumor growth) when compared with the group with free DOX (4.18 ± 0.71 times increase in tumor growth) at the same concentration of DOX and the MWCNT group (7.20 ± 2.04 times increase in tumor growth) with the same concentration of FA-targeted MWCNTs. On day 30, the tumor tissues were harvested, and the tumor tissues in the DOX/MWCNT group were smaller than all the other tumors (Figure S8). These results indicate that DOX/MWCNT NCs have an obvious tumor suppression effect, likely due to the PEGylation-enabled prolonged circulation time and the FA-mediated targeted delivery of DOX into the tumor. Furthermore, mouse weight after treatment with FA-targeted MWCNTs or DOX/MWCNT NCs was similar to mouse weight in the control group, whereas weight slowly increased for the mice treated with free DOX. This result indicates that FA-targeted MWCNTs decrease DOX-induced toxic side effects (Figure 4(b)).

### 3.9. Histological examination

**Figure 5.**
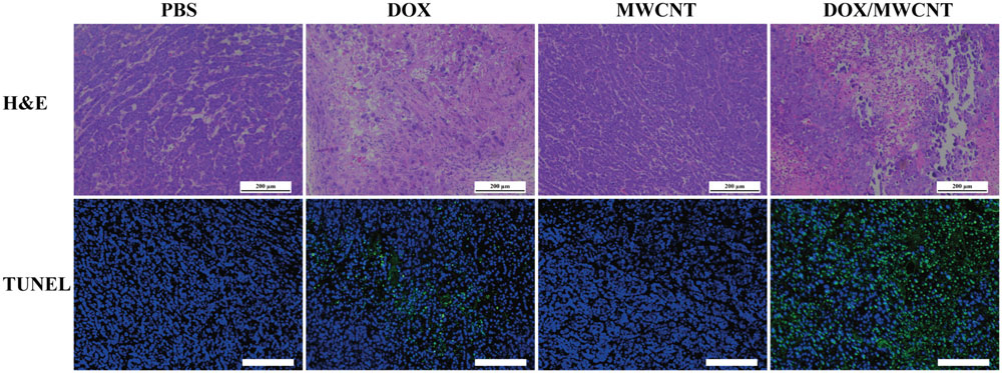
H&E staining and TUNEL staining of tumor sections after the tumor-bearing mice were treated differently according to grouping. The scale bar shown in each panel represents 100 μm.

The H&E staining and TUNEL staining of tumor sections were further performed to confirm the chemotherapeutic efficacy of tumors post-treatment (Figure 5). The H&E staining (Figure 5) indicated that the tumors treated with FA-targeted MWCNTs alone exhibited the well-shaped morphology of tumor cells similar to that of tumors without treatment (control group). The tumors treated with free DOX displayed a smaller necrosis area than the tumor section receiving injections of DOX/MWCNTs.

Figure 5 also shows that only a very small number of cells from the tumors in the control and MWCNT groups were positively stained. However, the cells from the tumors in the DOX group displayed a larger number of positively stained apoptotic cells (brown spots), and the cells from the tumors in the DOX/MWCNT group exhibited the largest number of positively stained apoptotic cells. The percentages of apoptotic cells in the tumor section from the control, DOX, MWCNT, and DOX/MWCNT groups were 12.5, 42.5, 9.1, and 73.3%, respectively (Figure S9).

### 3.10. Biodistribution study

The biodistribution of DOX in the major organs of mice was measured 12 h after an injection of DOX/MWCNTs (Figure S10). As expected for DOX/MWCNT NCs, the tumor uptake of DOX was 10.48% of the injected dose per gram of tissue (% ID/g), which is higher than previously reported (Liu et al., 2009). This increase is likely due to the PEGylation of DOX/MWCNT NCs, enabling them to escape capture by the RES and to enrich the tumor region via a passive targeting pathway based on the enhanced permeability and retention (EPR) effect (Liu et al., 2014); in contrast, NCs specifically aggregated in the tumor region via the FA-mediated active targeting pathway, as demonstrated in the *in vitro* studies. Compared with the DOX in tumors, less DOX accumulated in the heart (5.99% ID/g) and kidney (1.50% ID/g), and more DOX accumulated in RES organs, including the liver (20.15% ID/g) and spleen (7.49% ID/g). No obvious organ toxicity was found in the main organs of mice injected with DOX/MWCNT NCs (Figure S11) compared with that found in the main organs of control mice. Despite the high RES capture, DOX/SWCNT NCs show no hepatic or splenic toxicity (Liu et al., 2007).

## 4. Conclusion

In summary, DOX/MWCNT NCs were generated as a versatile nanoplatform for the chemotherapy of tumors. Acid-treated MWCNTs were grafted with PEI, which can be easily modified with the PEGylation targeting ligand FA and the fluorescent probe FI and can be acetylated by using acetic anhydride. The multifunctional FA-targeted MWCNTs combine the characteristics of exceptional colloidal stability, good biocompatibility, and high affinity for FAR-overexpressing cancer cells. After loading with an abundance of DOX molecules, DOX/MWCNT NCs were formed, and they displayed strong chemotherapeutic performance against cancer cells *in vitro* and tumors *in vivo*. The DOX/MWCNT NCs decreased side effects (e.g. cardiotoxicity) and increased the anti-tumor effect via exquisite targeted drug delivery and may be used as a versatile theranostic platform in translational medicine.

## Supplementary Material

Supplementary Figures

## References

[CIT0001] Assa F, Jafarizadeh-Malmiri H, Ajamein H, et al. (2017). Chitosan magnetic nanoparticles for drug delivery systems. Crit Rev Biotechnol 37:492. 27248312 10.1080/07388551.2016.1185389

[CIT0002] Bhirde AA, Patel V, Gavard J, et al. (2009). Targeted killing of cancer cells *in vivo* and *in vitro* with EGF-directed carbon nanotube-based drug delivery. ACS Nano 3:307–16. 19236065 10.1021/nn800551s PMC2665730

[CIT0003] Cao X, Tao L, Wen S, et al. (2015). Hyaluronic acid-modified multiwalled carbon nanotubes for targeted delivery of doxorubicin into cancer cells. Carbohydr Res 405:70–7. 25500334 10.1016/j.carres.2014.06.030

[CIT0004] Chłopek J, Czajkowska B, Szaraniec B, et al. (2006). *In vitro* studies of carbon nanotubes biocompatibility. Carbon 44:1106–11.

[CIT0005] Du C, Zhao J, Fei J, et al. (2013). Assembled microcapsules by doxorubicin and polysaccharide as high effective anticancer drug carriers. Adv Healthc Mater 2:1246. 23554398 10.1002/adhm.201200414

[CIT0006] Feazell RP, Nakayama-Ratchford N, Dai H, Lippard SJ (2007). Soluble single-walled carbon nanotubes as longboat delivery systems for platinum(IV) anticancer drug design. J Am Chem Soc 129:8438–9. 17569542 10.1021/ja073231f PMC2505197

[CIT0007] Hai W, Agarwal P, Zhao S, et al. (2015). A biomimetic hybrid nanoplatform for encapsulation and precisely controlled delivery of theranostic agents. Nat Commun 6:10081. 26621191 10.1038/ncomms10081 PMC4686774

[CIT0008] Hersam MC (2008). Progress towards monodisperse single-walled carbon nanotubes. Nature Nanotech 3:387–94. 10.1038/nnano.2008.135 18654561

[CIT0009] Hu Y, Wang R, Wang S, et al. (2016). Multifunctional Fe3O4 @ Au core/shell nanostars: a unique platform for multimode imaging and photothermal therapy of tumors. Sci Rep 6:28325. 27325015 10.1038/srep28325 PMC4914846

[CIT0010] Hu Y, Yang J, Wei P, et al. (2015). Facile synthesis of hyaluronic acid-modified Fe3O4/Au composite nanoparticles for targeted dual mode MR/CT imaging of tumors. J Mater Chem B 3:9098–108. 10.1039/c5tb02040a 32263123

[CIT0011] Huang P, Xu C, Lin J, et al. (2011). Folic acid-conjugated graphene oxide loaded with photosensitizers for targeting photodynamic therapy. Theranostics 1:240–50. 21562631 10.7150/thno/v01p0240 PMC3092447

[CIT0012] Kesharwani P, Jain K, Jain NK (2014). Dendrimer as nanocarrier for drug delivery. Prog Polym Sci 39:268–307.

[CIT0013] Lacerda L, Bianco A, Prato M, Kostarelos K (2006). Carbon nanotubes as nanomedicines: from toxicology to pharmacology. Adv Drug Deliv Rev 58:1460–70. 17113677 10.1016/j.addr.2006.09.015

[CIT0014] Li J, Hu Y, Yang J, et al. (2015). Facile synthesis of folic acid-functionalized iron oxide nanoparticles with ultrahigh relaxivity for targeted tumor MR imaging. J Mater Chem B 3:5720–30. 10.1039/c5tb00849b 32262568

[CIT0015] Li J, Zheng L, Cai H, et al. (2013). Polyethyleneimine-mediated synthesis of folic acid-targeted iron oxide nanoparticles for *in vivo* tumor MR imaging. Biomaterials 34:8382–92. 23932250 10.1016/j.biomaterials.2013.07.070

[CIT0016] Liu H, Wang H, Xu Y, et al. (2014). Synthesis of PEGylated low generation dendrimer-entrapped gold nanoparticles for CT imaging applications. Nanoscale 6:4521–6. 24647803 10.1039/c3nr06694k

[CIT0017] Liu W, Wen S, Shen M, Shi X (2014). Doxorubicin-loaded poly(lactic-co-glycolic acid) hollow microcapsules for targeted drug delivery to cancer cells. New J Chem 38:3917–24.

[CIT0018] Liu Z, Cai W, He L, et al. (2007). *In vivo* biodistribution and highly efficient tumour targeting of carbon nanotubes in mice. Nat Nanotechnol 2:47–52. 18654207 10.1038/nnano.2006.170

[CIT0019] Liu Z, Fan AC, Rakhra K, et al. (2009). Supramolecular stacking of doxorubicin on carbon nanotubes for *in vivo* cancer therapy. Angew Chem Int Ed Engl 48:7668–72. 19760685 10.1002/anie.200902612 PMC2824548

[CIT0020] Liu Z, Sun X, Nakayama-Ratchford N, Dai H (2007). Supramolecular chemistry on water-soluble carbon nanotubes for drug loading and delivery. ACS Nano 1:50–6. 19203129 10.1021/nn700040t

[CIT0021] Low PS, Henne WA, Doorneweerd DD (2008). Discovery and development of folic-acid-based receptor targeting for imaging and therapy of cancer and inflammatory diseases. Acc Chem Res 41:120–9. 17655275 10.1021/ar7000815

[CIT0022] Movassaghian S, Merkel OM, Torchilin VP (2015). Applications of polymer micelles for imaging and drug delivery. Wires Nanomed Nanobiotechnol 7:691. 10.1002/wnan.1332 25683687

[CIT0023] Petersen EJ, Huang Q, Weber J, Walter J (2008a). Bioaccumulation of radio-labeled carbon nanotubes by Eisenia foetida. Environ Sci Technol 42:3090–5. 18497171 10.1021/es071366f

[CIT0024] Petersen EJ, Huang Q, Weber WJ. Jr. (2008b). Ecological uptake and depuration of carbon nanotubes by Lumbriculus variegatus. Environ Health Perspect 116:496. 18414633 10.1289/ehp.10883 PMC2290976

[CIT0025] Prato M, Kostarelos K, Bianco A (2008). Functionalized carbon nanotubes in drug design and discovery. Acc Chem Res 41:60–8. 17867649 10.1021/ar700089b

[CIT0026] Qu Q, Ma X, Zhao Y (2015). Targeted delivery of doxorubicin to mitochondria using mesoporous silica nanoparticle nanocarriers. Nanoscale 7:16677. 26400067 10.1039/c5nr05139h

[CIT0027] Shen M, Wang SH, Shi X, et al. (2009). Polyethyleneimine-mediated functionalization of multiwalled carbon nanotubes: synthesis, characterization, and *in vitro* toxicity assay. J Phys Chem C 113:3150–6.

[CIT0028] Shi Kam NW, Jessop TC, Wender PA, Dai H (2004). Nanotube molecular transporters: internalization of carbon nanotube-protein conjugates into mammalian cells. J Am Chem Soc 126:6850–1. 15174838 10.1021/ja0486059

[CIT0029] Shi X, Wang SH, Shen M, et al. (2009). Multifunctional dendrimer-modified multiwalled carbon nanotubes: synthesis, characterization, and *in vitro* cancer cell targeting and imaging. Biomacromolecules 10:1744–50. 19459647 10.1021/bm9001624

[CIT0030] Sun C, Sze R, Zhang M (2006). Folic acid-PEG conjugated superparamagnetic nanoparticles for targeted cellular uptake and detection by MRI. J Biomed Mater Res 78A:550–7. 10.1002/jbm.a.30781 16736484

[CIT0031] Tahover E, Patil YP, Gabizon AA (2015). Emerging delivery systems to reduce doxorubicin cardiotoxicity and improve therapeutic index: focus on liposomes. Anticancer Drugs 26:241. 25415656 10.1097/CAD.0000000000000182

[CIT0032] Wang S, Wu Y, Guo R, et al. (2013). Laponite nanodisks as an efficient platform for doxorubicin delivery to cancer cells. Langmuir 29:5030–6. 23419072 10.1021/la4001363

[CIT0033] Wang Y, Cao X, Guo R, et al. (2011). Targeted delivery of doxorubicin into cancer cells using a folic acid–dendrimer conjugate. Polym Chem 2:1754–60.

[CIT0034] Wen S, Liu H, Cai H, et al. (2013). Targeted and pH‐responsive delivery of doxorubicin to cancer cells using multifunctional dendrimer‐modified multi‐walled carbon nanotubes. Adv Healthcare Mater 2:1267–76. 10.1002/adhm.201200389 23447549

[CIT0035] Zerda A, Bodapati S, Teed R, et al. (2012). Family of enhanced photoacoustic imaging agents for high-sensitivity and multiplexing studies in living mice. ACS Nano 6:4694–701. 22607191 10.1021/nn204352r PMC3397693

[CIT0036] Zhu J, Zheng L, Wen S, et al. (2014). Targeted cancer theranostics using alpha-tocopheryl succinate-conjugated multifunctional dendrimer-entrapped gold nanoparticles. Biomaterials 35:7635–46. 24927683 10.1016/j.biomaterials.2014.05.046

