# Supplementary material for: Stacking of doxorubicin on folic acid-targeted multiwalled carbon nanotubes for in vivo chemotherapy of tumors
Source: Drug Deliv. 2018 Oct 22;25(1):1607–16. doi: 10.1080/10717544.2018.1501120 (PMC6201812; doi:10.1080/10717544.2018.1501120)
Supplement: Supplementary Figures [file IDRD_A_1501120_SM8232.doc]

**Supporting Information**

**Stacking of doxorubicin on folic acid-targeted multiwalled carbon nanotubes for in vivo cancer therapy**

Yan Yan, ^a1^* Ruizhi Wang,^b1^Yong Hu,^c1^ Rongyue Sun,^b^ Tian Song,^b^ Xiangyang Shi, ^c^* Shimeng Yin,^b^*

^a^ Department of Obstetrics, Shanghai First Maternity and Infant Hospital, Tongji University School of Medicine, Shanghai 201204, P. R. China

^b^ Department of Radiology, Huadong Hospital, Fudan University, Shanghai 200040, P. R. China

^c^ College of Chemistry, Chemical Engineering and Biotechnology, Donghua University, Shanghai 201620, P. R. China

_________________________________________________________________

* The corresponding authors’ E-mail: xshi@dhu.edu.cn (X. Shi), yinfang101@126.com (S. Yin), and yanyan.doc@163.com (Y. Yan).

^1^ Authors contributed equally to this work.


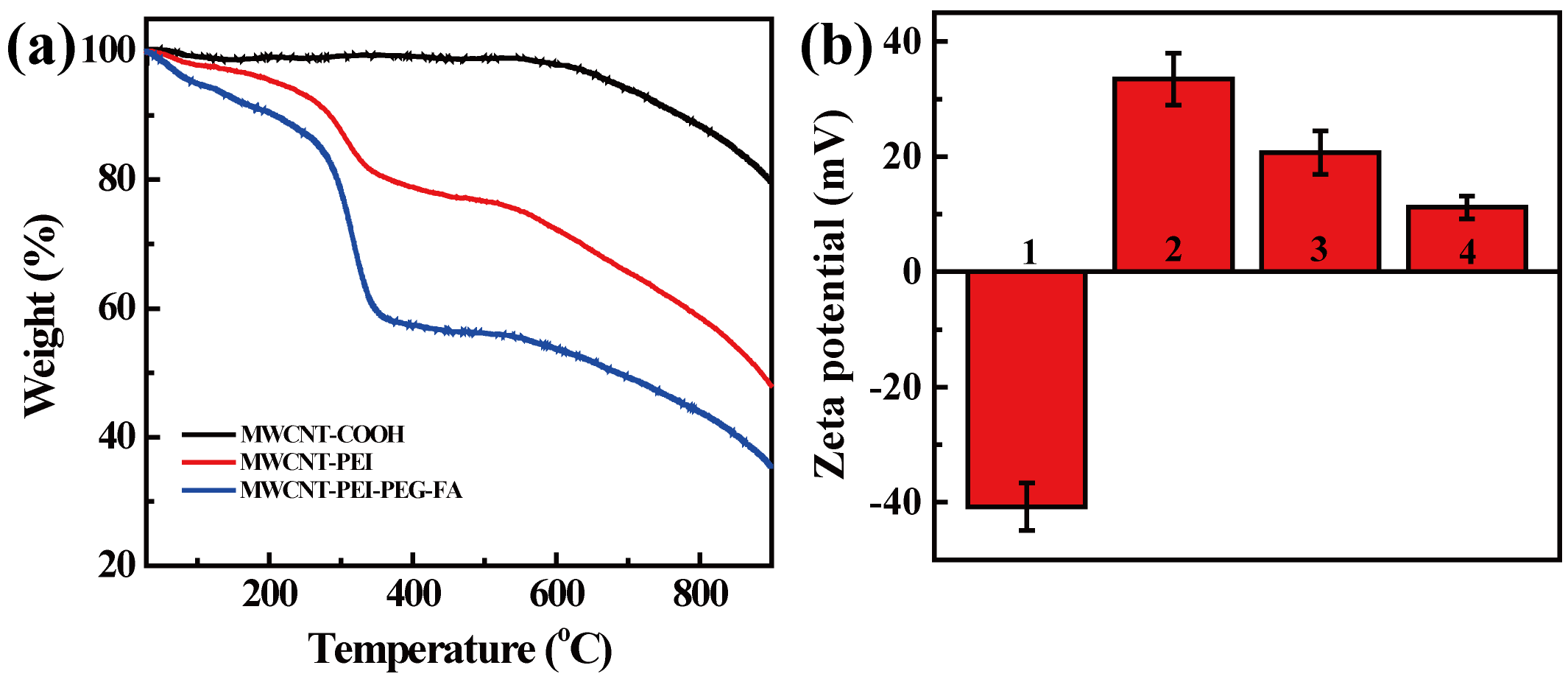


**Figure S1.** (a) TGA curves of MWCNT-COOH, MWCNT-PEI, and MWCNT-PEI-PEG-FA. (b) Zeta potentials of MWCNT-COOH (1), MWCNT-PEI (2), MWCNT-PEI-FI-PEG-FA (3), and MWCNT-PEI.Ac-FI-PEG-FA (4).

**Figure S2.** UV-Vis spectra of MWCNT-PEI, MWCNT-PEI-PEG-FA, and MWCNT-PEI-FI-PEG-FA dispersed into water, respectively.


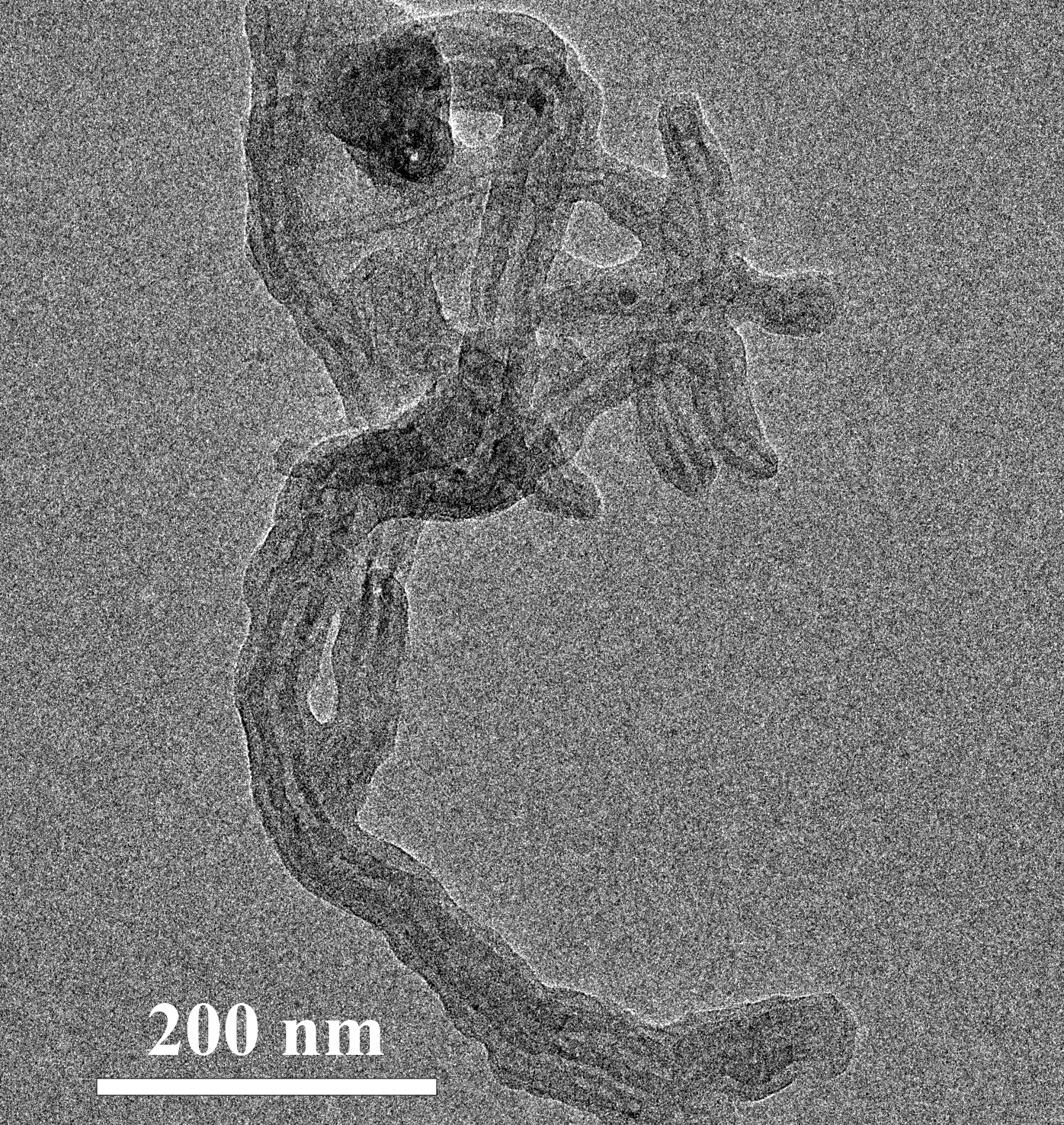


**Figure S3.** TEM image of MWCNT-PEI.Ac-FI-PEG-FA.

**Figure S4.** UV-Vis spectrum of DOX/MWCNT NCs dispersed into water.

**Figure S5.** Release of DOX from DOX/MWCNT NCs (2 mg/mL) at different pH values.


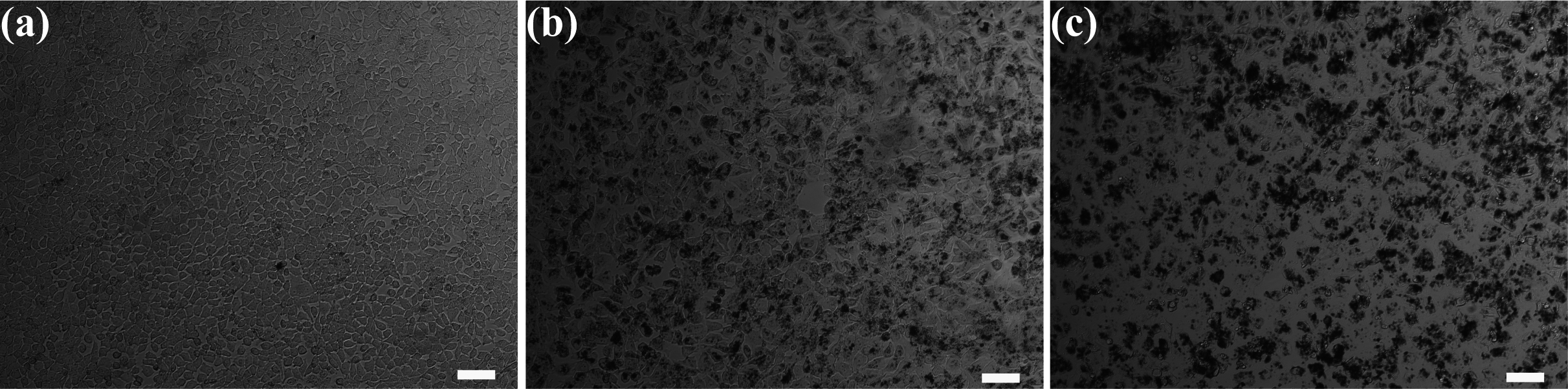


**Figure S6.** The photos of HeLa cells treated with PBS (a), MWCNT-PEI.Ac-FI-PEG-FA at the concentration of 8.4 mg/L (b), and DOX/MWCNT at the DOX concentration of 4 mg/L for 24 h. The scale bar represent 100 μm.

**Figure S7.** CCK-8 assays to evaluate the viability of HeLa-HFAR and HeLa-LFAR cells after 2 h of treatment with DOX/MWCNT NCs ([DOX] = 2 mg/L). The medium was replaced with DOX-free fresh medium, and the cultivation of the cells continued for 48 h.


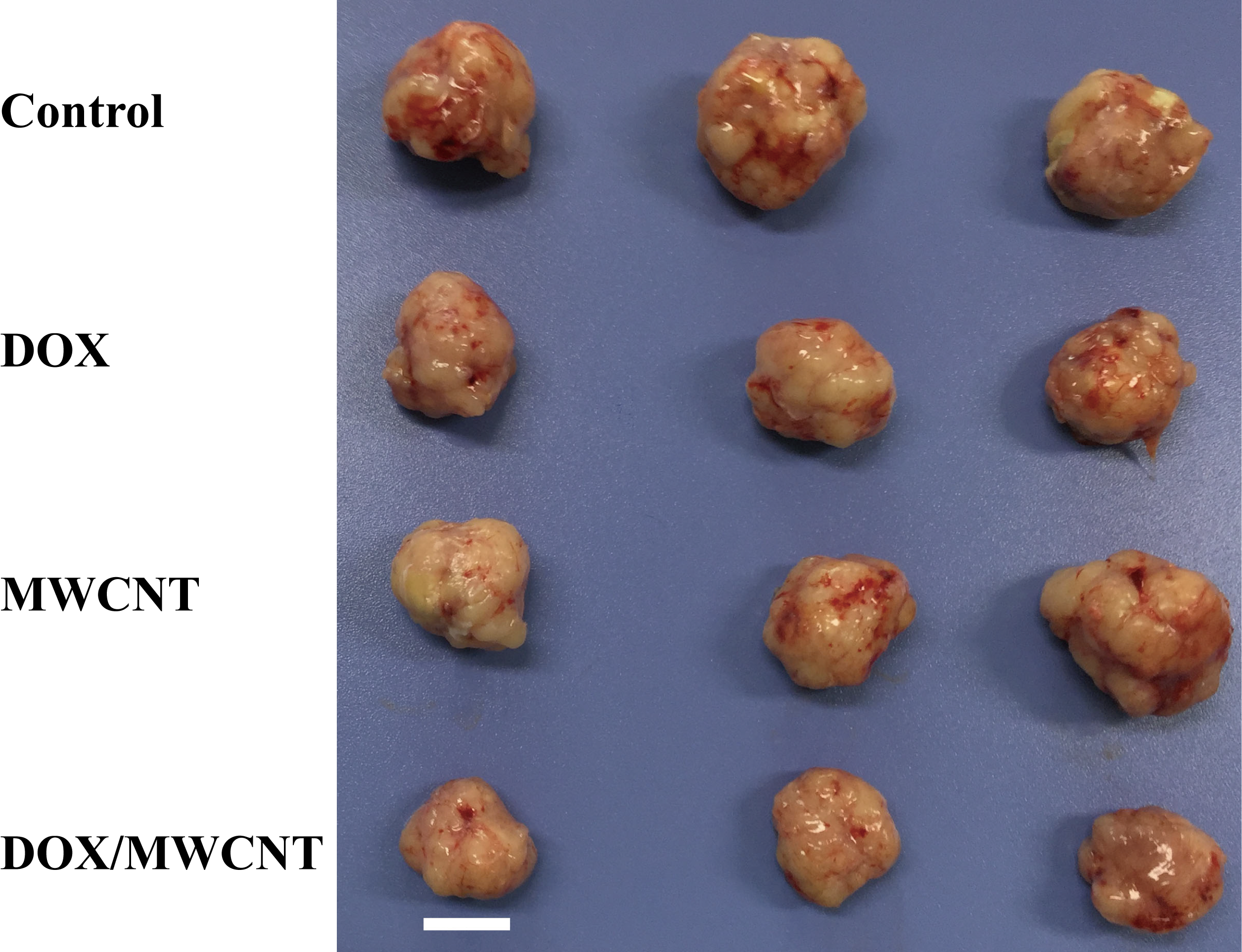


**Figure S8.** Photographs of the harvested tumors from different groups at 30 days after the indicated treatment. The scale bar represents 1 cm.

**Figure S9.** Apoptosis rate of tumor cells after different treatments by quantification of the TUNEL-positive tumor cells in tumor sections.

**Figure S10.** Biodistribution of DOX in major organs of mice measured 12 h after injection of DOX/MWCNTs.


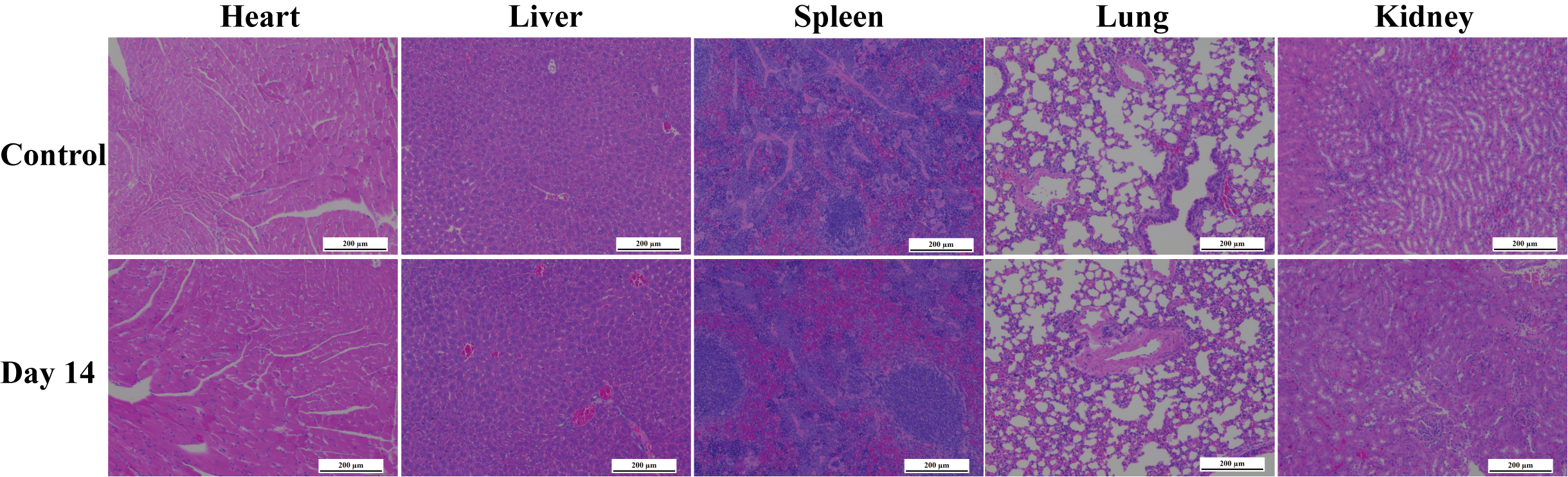


**Figure S11.** H&E staining of main organ sections of the healthy mice after injected with DOX/MWCNT NCs for 14 days. The scale bar shown in each panel represents 200 μm.
